# Supplementary material for: A Spanish Survey on the Perioperative Use of Antimicrobials in Small Animals
Source: Animals (Basel). 2023 Jul 31;13(15):2475. doi: 10.3390/ani13152475 (PMC10417378; doi:10.3390/ani13152475)
Supplement: Supplementary file 1 [file animals-13-02475-s001.zip › animals-2509496-supplementary.pdf]

Table S1. Demographic data of survey participants (N = 558).

Variable	Category	Respondents (%)	Respondents (n)
Gender	Female	55.4	249
	Male	44.6	309
University where respondent obtained their Veterinary degree	Faculty of Barcelona (UAB)	0.9	5
	Faculty of Córdoba	16.7	93
	Faculty of Las Palmas de Gran Canaria	8.2	46
	Faculty of Cáceres	2.3	13
	Faculty of León	3.9	22
	Faculty of Lugo	7.9	44
	Faculty of Madrid (UCM)	7.3	41
	Private University Alfonso X El Sabio	21.7	121
	Faculty of Murcia	5.2	29
	Private University Cardenal Herrera CEU Valencia	5.6	31
	Private University Católica de Valencia San Vicente Mártir	3.4	19
	Faculty of Zaragoza	16.8	94
Postgraduate training in surgery	No surgical training	57.7	322
	ECVS/ACVS Diploma	2.7	15
	Postgraduate masters	6.5	36
	Postgraduate course	28.0	156
	PhD related to small animal surgery	5.2	29
Percentage of activity of the veterinary clinic dedicated to small animals (%)	≤ 75	3.0	17
	> 75	97.0	541
Annual average percentage dedicated to small animal surgery (%)	≤ 75	79.9	446
	> 75	20.1	112
Type of veterinary centre	Public	7.2	40
	Private	92.8	518
Autonomous communities of Spain where you currently work	Andalucía	8.4	47
	Aragón	5.4	30
	Principado de Asturias	3.0	17
	Islas Baleares	4.1	23
	Canarias	4.7	26
	Cantabria	1.6	9
	Castilla La Mancha	1.8	10
	Castilla y León	5.9	33
	Cataluña	17.0	95
	Comunidad Valenciana	6.8	38

	Extremadura	1.8	10
	Galicia	6.6	37
	La Rioja	1.3	7
	Comunidad de Madrid	23.5	131
	Comunidad Foral de Navarra	1.1	6
	País Vasco	5.0	28
	Región de Murcia	2.0	11
Years of experience		14.0 *	(7.0-24.0)**
Total number of veterinarians		3.0 *	(2.0-7.5)**
Surgeons out of the total number of veterinarians in the center.		2.0*	(2.0-3.0)**
Total number of veterinary assistants		2.0*	(1.0-3.0)**

*Median; **IQR

Table S2. Number of respondents, percentage and median score of veterinarians who ranked routes and time of perioperative antimicrobial administration.

Category	Before surgery n (%)	During surgery n (%)	After surgery n (%)	Postoperatory n (%)	Administration route of not used n (%)
Subcutaneous	312 (55.9)	26 (4.7)	106 (19.0)	41 (7.3)	73 (13.1)
Intravenous	178 (31.9)	247 (44.3)	17 (3.0)	9 (1.6)	107 (19.2)
Intramuscular	105 (18.8)	18 (3.2)	60 (10.8)	28 (5.0)	347 (62.2)
Oral	61 (10.9)	0 (0.0)	8 (1.4)	469 (84.1)	20 (3.6)
Topical	41 (7.3)	3 (0.5)	50 (9.0)	163 (29.2)	301 (53.9)

Table S3. Number of respondents, percentage and median score of veterinarians who ranked the information source for determination of the dosage recommendations of perioperative antimicrobials.

Category	1 (%)	2 (%)	3 (%)	4 (%)	n	Median	25	75
Vademecum/Prospectus	102 (21.2)	85 (17.7)	140 (29.1)	154(32)	481	3.0	2.0	4.0
Books and user guidelines	22 (4.6)	33 (6.9)	127 (26.5)	298 (62.1)	480	4.0	3.0	4.0
Conference proceedings	83 (17.4)	136 (28.6)	143 (30)	114 (23.9)	476	3.0	2.0	3.0
Scientific articles	95 (19.6)	119 (24.6)	120 (24.8)	150 (31)	484	3.0	2.0	4.0

Table S4. Univariate and multivariate logistic regression model of use of perioperative antimicrobials for ovariohysterectomy and orchiectomy in dogs and cats and demographic data.

Variable		Use of antimicrobials		Univariate				Multivariate			
		Low-frequency use (never, rarely, sometimes)	High- frequency use (usually and always)	OR	95%CI Lower	95%CI upper	p-value	adjusted-OR	95%CI low	95%CI Upper	p-value
**Gender**	Female	128	167			1			-		
	Male	103	137	1.02	0.72	1.44	0.91		-		
**Surgical training**	Non surgical postgraduate training	149	158			1			1		
	Surgical postgraduate training	82	146	1.68	1.18	2.38	0.004	1.65	1.14	2.38	0.008
**Percentage dedicated to surgery in small animals in annual average (%)**	≤75	211	260			1			-		
	>75	20	44	1.78	1.02	3.23	0.042		-		
**Surgeons out of the total number of veterinarians in the center**	≤2	152	162			1			-		
	>2	74	140	1.77	1.24	2.54	0.002		-		
**Total number of vets**	≤3	138	130			1			1		
	>3	88	171	2.06	1.45	2.93	<0.001	1.75	1.22	2.52	0.002
**Years of experience**	≤14	90	181	2.33	1.61	3.23	<0.001	2.12	1.48	3.04	0.001
	>14	141	123			1			1		

Use of postoperative antimicrobials in ovariohysterectomy in dogs

Variable		Use of antimicrobials		Univariate				Multivariate			
		Low-frequency use (never, rarely, sometimes)	High- frequency use (usually and always)	OR	95%CI Lower	95%CI Upper	p-value	adjusted-OR	95%CI low	95%CI Upper	p-value
**Gender**	Female	55	241	1.92	1.28	2.85	0.002		-		

	Male	73	167	1				-			
**Surgical training**	Non surgical postgraduate training	51	256	2.56	1.7	3.84	<0.001	2.20	1.43	3.45	<0.001
	Surgical postgraduate training	77	152	1				1			
**Percentage dedicated to surgery in small animals in annual average (%)**	≤75	100	372	2.86	1.69	5.00	<0.001	2.19	1.24	3.86	0.007
	>75	28	36	1				1			
**Surgeons out of the total number of veterinarians in the center**	≤2	56	260	2.85	1.69	5.00	<0.001	2.01	1.32	3.06	0.001
	>2	70	143	1				1			
**Total number of vets**	≤3	49	220	1.88	1.26	2.85	0.002	-			
	>3	77	182	1				-			
**Years of experience**	≤14	66	205	1				-			
	>14	62	203	1.05	0.71	1.57	0.795	-			

Use of preoperative antimicrobials in ovariohysterectomy in cats

**Variable**		**Use of antimicrobials**		**Univariate**				**Multivariate**			
		**Low-frequency use (never, rarely, sometimes)**	**High- frequency use (usually and always)**	**OR**	**95%CI Lower**	**95%CI Upper**	**p-value**	**adjusted-OR**	**95%CI low**	**95%CI Upper**	**p-value**
**Gender**	Female	131	164	1.02	0.72	1.42	0.915	-			
	Male	105	129	1				-			
**Surgical training**	Non surgical postgraduate training	150	154	1				1			

	Surgical postgraduate training	86	139	1.57	1.11	2.23	0.011	2.16	1.51	3.09	<0.001
**Percentage dedicated to surgery in small animals in annual average (%)**	≤ 75	215	250	1				-			
	>75	21	43	1.76	1.01	3.06	0.045	-			
**Surgeons out of the total number of veterinarians in the center**	≤2	154	154	1				-			
	>2	77	137	1.78	1.24	2.54	0.002	-			
**Total number of vets**	≤3	142	119	1				1			
	>3	89	170	2.28	1.60	3.25	<0.001	2.16	1.51	3.09	<0.001
**Years of experience**	≤ 14	90	178	2.5	1.78	3.57	<0.001	-			
	>14	146	115	1				-			

Use of postoperative antimicrobials in ovariohysterectomy in cat

Variable		Use of antimicrobials		Univariate				Multivariate			
		Low-frequency use (never. rarely. sometimes)	High- frequency use (usually and always)	OR	95%CI Lower	95%CI Upper	p-value	adjusted-OR	95%CI low	95%CI Upper	p-value
**Gender**	Female	78	217	1.66	1.03	2.17	0.033	-			
	Male	82	152	1				-			
**Surgical training**	Non surgical postgraduate training	67	235	2.44	1.67	3.57	<0.001	2.44	1.67	3.57	<0.001
	Surgical postgraduate training	93	134	1				1			

**Percentage dedicated to surgery in small animals in annual average (%)**	≤75	128	337	2.63	1.56	4.54	<0.001	2.12	2.21	3.70	<0.001
	>75	32	32	1				1			
**Surgeons out of the total number of veterinarians in the center**	≤2	76	232	1.85	1.26	2.70	0.001	-			
	>2	81	133	1				-			
**Total number of vets**	≤3	64	197	1.72	1.16	2.5	0.006	-			
	>3	92	167	1				-			
**Years of experience**	≤14	88	178	1				-			
	>14	72	191	1.31	0.90	1.90	0.154	-			

Use of preoperative antimicrobials in orchiectomy in dogs

**Variable**		**Use of antimicrobials**		**Univariate**				**Multivariate**			
		**Low-frequency use (never. rarely. sometimes)**	**High- frequency use (usually and always)**	**OR**	**95%CI Lower**	**95%CI Upper**	**p-value**	**adjusted-OR**	**95%CI low**	**95%CI Upper**	**p-value**
**Gender**	Female	145	156	1.03	0.73	1.44	0.853	-			
	Male	119	124	1				-			
**Surgical training**	Non surgical postgraduate training	160	152	1				-			
	Surgical postgraduate training	104	128	1.29	0.92	1.82	0.137	-			
**Percentage dedicated to surgery in small animals in annual average (%)**	≤75	175	146	1				-			
	>75	84	132	1.39	0.82	2.37	0.222	-			

**Surgeons out of the total number of veterinarians in the center**	≤2	106	216	1				-			
	>2	87	128	1.88	1.33	2.67	<0.001	-			
**Total number of vets**	≤3	158	118	1				1			
	>3	101	159	2.10	1.49	2.98	<0.001	1.92	1.35	2.74	<0.001
**Years of experience**	≤14	103	174	2.56	1.81	3.57	<0.001	2.34	1.65	3.34	<0.001
	>14	161	106	1				1			

Use of postoperative antimicrobials in orchiectomy in dogs

**Variable**		**Use of antimicrobials**		**Univariate**				**Multivariate**			
		**Low-frequency use (never. rarely. sometimes)**	**High- frequency use (usually and always)**	**OR**	**95%CI Lower**	**95%CI Upper**	**p-value**	**adjusted-OR**	**95%CI low**	**95%CI Upper**	**p-value**
**Gender**	Female	98	204	1.42	1.00	2.00	0.052	-			
	Male	98	144	1				-			
**Surgical training**	Non surgical postgraduate training	166	150	2.08	1.45	2.94	<0.001	1.89	1.30	2.70	<0.001
	Surgical postgraduate training	106	126	1				1			
**Percentage dedicated to surgery in small animals in annual average (%)**	≤75	159	321	2.77	1.63	4.47	<0.001	2.32	1.35	4.00	0.002
	>75	37	27	1				1			
**Surgeons out of the total number of veterinarians in the center**	≤2	106	216	1.39	0.97	2.03	0.075	-			
	>2	87	128	1				-			

**Total number of vets**	≤3	89	185	1.35	0.94	1.92	0.100	-			
	>3	103	159	1				-			
**Years of experience**	≤14	105	170	1				-			
	>14	91	178	1.21	0.85	1.71	0.291	-			

Use of preoperative antimicrobials in orchiectomy in cats

**Variable**		**Use of antimicrobials**		**Univariate**				**Multivariate**			
		**Low-frequency use (never. rarely. sometimes)**	**High- frequency use (usually and always)**	**OR**	**95%CI Lower**	**95%CI Upper**	**p-value**	**adjusted-OR**	**95%CI low**	**95%CI Upper**	**p-value**
**Gender**	Female	152	151	1				-			
	Male	121	124	1.03	0.74	1.44	0.856	-			
**Surgical training**	Non surgical postgraduate training	168	148	1				-			
	Surgical postgraduate training	105	127	1.37	0.98	1.93	0.068	-			
**Percentage dedicated to surgery in small animals in annual average (%)**	≤75	251	233	1				1			
	>75	22	42	2.05	1.19	3.55	0.01	2.31	1.32	4.04	0.003
**Surgeons out of the total number of veterinarians in the center**	≤2	177	148	1				-			
	>2	90	126	1.67	1.18	2.37	0.04	-			
**Total number of vets**	≤3	156	122	1				-			
	>3	112	149	1.70	1.21	2.40	0.002	-			
**Years of experience**	≤14	113	164	2.08	1.49	2.94	<0.001	2.22	1.56	3.12	<0.001
	>14	160	111	1				1			

Use of postoperative antimicrobials in orchiectomy in cats											
**Variable**		**Use of antimicrobials**		**Univariate**				**Multivariate**			
		**Low-frequency use (never. rarely. sometimes)**	**High- frequency use (usually and always)**	**OR**	**95%CI Lower**	**95%CI Upper**	**p-value**	**adjusted-OR**	**95%CI low**	**95%CI Upper**	**p-value**
**Gender**	Female	163	141	1.35	0.96	1.92	0.080	-			
	Male	149	95	1				-			
**Surgical training**	Non surgical postgraduate training	166	148	1.47	1.05	2.08	0.026	1.45	1.02	2.08	0.040
	Surgical postgraduate training	146	88	1				-			
**Percentage dedicated to surgery in small animals in annual average (%)**	≤75	268	216	1.78	1.02	3.12	0.044	-			
	>75	44	20	1				-			
**Surgeons out of the total number of veterinarians in the center**	≤2	178	148	1.25	0.88	1.78	0.215	-			
	>2	129	86	1				-			
**Total number of vets**	≤3	144	134	1.51	1.07	2.12	0.016	1.44	1.02	2.04	0.043
	>3	162	99	1				1			
**Years of experience**	≤14	156	120	1.04	0.73	0.73	0.844	-			
	>14	156	116	1				-			
